# Recycling electro-coagulated sludge from textile wastewater treatment plants as an adsorbent for the adsorptions of fluoride in an aqueous solution

**DOI:** 10.1016/j.heliyon.2021.e07281

**Published:** 2021-06-11

**Authors:** Tadele Assefa Aragaw

**Affiliations:** Faculty of Chemical and Food Engineering, Bahir Dar Institute of Technology, Bahir Dar University, Bahir Dar, Ethiopia

**Keywords:** EC sludge, Adsorption, Characterization, Defluoridation, Equilibrium

## Abstract

This research investigated the high content of iron-based materials from recycled electro-coagulated (EC) sludge for the adsorptive removal of fluoride, and the properties of the material were characterized. The thermal activation of EC sludge in which the unwanted impurity was removed by beneficiation and thermally activated at 500 °C, and was used for fluoride removal. Basic operating parameters (mixing time, adsorbent dosage, adsorbate concentration, solution pH, and temperature) were examined to evaluate the optimum de-fluoridation capacity (DC). The functional groups, the crystalline structure, and surface morphology of thermally treated and raw EC sludge were analyzed using FTIR, XRD, and SEM, respectively, and demonstrates that thermally activated EC sludge contains significant content of magnetite and hematite. The optimum DC was recorded as 5.12 mg of F^−^/gm with experimental conditions: mixing time = 20 min, adsorbent dosage = 0.3 gm/100 ml, initial fluoride concentration = 1 mg/L, and pH = 5 at the temperature of 353 K. The Langmuir isotherm model was fitted, and the capacity is calculated as 6.43 mg/g. The adsorption process follows the Pseudo-Second-order kinetic models. It can be concluded that the prepared adsorbents have excellent fluoride removal capacity, and EC sludge can be used as an alternative adsorbent for de-fluoridation.

## Highlights

• Iron-based oxides and hydroxides from the EC sludge were recovered and prepared for fluoride ion adsorption.
• EC sludge as an iron-based adsorbent was synthesized by thermal activation at 500 °C.
• Iron oxide adsorbents could efficiently remove fluoride ions from synthetically prepared water solutions.
• The adsorption of fluoride followed a Langmuir isotherm pseudo-Second-order kinetic model.
• The prepared adsorbents were regenerated in an aqueous solution and the reusability efficiency was up to the 4th cycle.

## 1 Introduction

Fluoride ions have occurred in the natural waters. It is a dynamic micronutrient in humans for dental and facilitates the mineralization of hard tissues if taken beyond the proposed range of concentrations. The upper limit of fluoride concentration in drinking water is recorded as a value of 1.5 mg/L, as per the World Health Organization (WHO) guidelines [1]. A higher concentration than 1.5 mg/L can lead to fluorosis (dental and skeletal) and numerous types of neurological harm for humans. Worldwide, approximately 30% of drinking water from groundwater sources is fluoride-rich [1]. As fluorosis is an incurable and irreversible disease, prevention by the de-fluoridation technique is the best option. Thus, it is a must to develop cost-effective, eco-friendly alternative non-conventional adsorbents for fluoride ion removal from an aqueous solution.

Several defluoridation techniques have been accounted for the removal of fluoride from drinking water, such as adsorption [2], chemical precipitation [3], ion exchange [4], reverse osmosis [5], nanofiltration [6], electrocoagulation [7], and electrodialysis [8]. Adsorptive removal of fluoride ions in water by conventional as well as non-conventional adsorbents has become more fascinating because of their easy operation, economics, and more efficiency than the other DC techniques [9]. Different adsorbents, for the removal fluoride ions from water and wastewater, such as alumina-based [10], clay-based [11], agricultural biomass [12], zeolite [13], calcium-based [14], carbon-based [15], and biopolymer-based materials [16] were reportedly used. Moreover, iron oxides were studied as potential adsorbents [17] and catalysts [18] in addition to the pigmentation application in manufacturing industries [19]. Also, it is used for medical purposes applications [20]. Frequently used iron oxides as an adsorbent are a hematite (α-Fe2O3), maghemite (γ-Fe2O3), and magnetite (Fe3O4). These iron-based oxides can be produced by the oxidative transformations from iron hydroxide and/or oxy-hydroxide through thermal treatment at a certain temperature.

Conversely, the high production of sludge, from treatment plants, as waste and its consequent treatment are sensitive environmental problems. Hence, emerging research is required for the sustainable treatment of sludge as a value-added resource. For example, transforming them into adsorbents for contaminants removal. Electro-coagulated (EC) sludge could be altered into alternative value-added products, such as adsorbents, which is a favorable way for resource utilization and secondary pollution control as they are unused resources and also presents serious disposal problems. Thus, using recyclable EC sludge in the adsorbent preparation for resource utilization as well as environmental management were employed to be bi-functional aspects. Preparing an iron-based adsorbent from EC sludge is cost-effective alternative adsorbents, and has found a remarkable adsorption performance for the removal of fluoride ions. Because of their outstanding chemical properties, and thermal stability [21]. EC sludge is primarily composed of an iron oxide-hydroxide precipitate that is produced by the redox reaction during the treatment of wastewater. Most of the reports on the de-fluoridation capacities of iron-based adsorbents are based on commercial iron oxide and iron oxide composites that are directly purchased from the market. However, its cost may not be affordable and unsustainable to the community as compared with the prepared adsorbents from EC sludge. Thus, the direct use of raw EC sludge or modified adsorbent for different pollutant adsorption has received great attention in the fields of material recycling, resource utilization, and industrial wastewater management.

So far reports assured that the adsorbent modification through different techniques is essential for their versatility and cost-competitiveness, easy regeneration, and removal efficiency for removing organic, and inorganic pollutants from waters and wastewater treatment applications. For example, chitosan/glutaraldehyde modified adsorbent using ammonium hydroxide for dye removal [22]; a thiourea-formaldehyde modified titan yellow sorbent for magnesium ion removal [23]; the thiourea-formaldehyde resin as a magnetic sorbent with magnetite particles (as a precursor) modification for the removal of dyes [24]; sorbents from the sepia shell with urea-formaldehyde modification for anionic dye removal [25]; the magnetic alginate magnetic sorbents prepared through alkaline treatment in them with sodium alginate for the removal of crystal violet [26] are some of the reports.

This study aimed to prepare iron-based adsorbents from EC sludge in industrial wastewater treatment plants and utilize them for defluoridation from synthetically prepared sodium fluoride (Na) aqueous solutions.

The beneficiation and thermal treatment of the EC sludge were conducted for adsorbent preparation from EC sludge, and performance evaluation for fluoride removal were performed. The study anticipates defluoridation from synthetically prepared NaF solution as a modal fluoride in the drinking water. The raw and modified adsorbents were analyzed using Fourier transforms infrared spectroscopy (FTIR), X-ray diffraction (XRD), and Scanning electron microscope (SEM). The basic batch adsorption operation parameters were performed. Moreover, the linear fitting adsorption isotherm models, the kinetic models, and thermodynamic properties of the adsorption phenomenon were computed. Finally, the regeneration potentials of the prepared adsorbent were examined for up to six cycles.

## 2 Materials and methods

### 2.1 Sample collection and solution preparation

The EC sludge samples were collected from Bahir Dar Textile factory, Bahir Dar, Ethiopia wastewater treatment plant having an electrochemical unit process. Analytical grade Sodium fluoride (NaF) with a purity of 97 % was used for the preparation of fluoride solution. It was supplied by the Bahir Dar Institute of Technology and used without further purification. A stock fluoride solution (1000 mg/L) was prepared by dissolving 2.21 g of NaF in 1000 mL of double-distilled water for the subsequent batch experiments. The selection of NaF as a modal fluoride source is due to the reason that sodium fluoride is the most commonly found salt in drinking groundwater than other types of salt, and it is easily soluble with ambient conditions. Other metal fluorides like calcium and magnesium could form insoluble complexes relative to sodium [27]. The desired number of working solutions was prepared by dilution of the stock solutions for DC studies.

### 2.2 Adsorbent preparation

Collected EC sludge was pre-treated at 70 °C using an oven for the removal of moistures. The size reduction (crushing and milling) was conducted by using a jaw crusher and miller, respectively, and the grounded powder was sieved to pass less than 200 μm. The grounded and milled EC sludge were dispersed into distilled water for a maximum of 24 h using conical-shaped 250 ml glassware. This is essential to remove the impurities (such as floatable oils, dissolved solid particles, and specks of dust). After decanting the supernatant, the residual slurry was washed carefully until the suspended particle was removed, and dried at 70 °C to remove the moisture. The dried solids were grounded and milled again for thermal treatment. Adsorbents were prepared through thermal treatment at a temperature of 500 °C from size reduced and wet treated EC sludge according to previous works [28]. The water content of the studied EC sludge was reported as 5.2% and the loss was recorded up to 99.7 °C in the thermogravimetric analysis [29]. The thermal treatment was conducted using a muffled furnace (Nabertherm LT 3/11/P330) with a constant nitrogen flow rate at 10 ml/min for 3 h to produce maghemite and/or hematite iron nanoparticle.

### 2.3 Adsorbent characterization

Fourier transform infrared spectroscopy (FT-IR) spectral analysis of the adsorbents before and after fluoride treatment was analyzed using a JASCO-6600 spectrophotometer in the range of 400–4000 cm^−1^. The adsorbent was ground with a ceramic mortar and mixed with potassium bromide (KBr), and the pellets were formed by pressing using a mechanical press. The crystalline and phase changes of untreated and thermally activated (500 °C) EC sludge-based adsorbents were examined using an X-ray diffractometer (XRD) (model: (SHIMADZU, MAXima-X XRD-7000). The CuK radiation, a voltage of 40.0 kV, the continuous scan range of 10–80 instrumental parameters were employed. The prepared adsorbent morphology was examined for the untreated and thermally activated (500 °C) EC sludge using a scanning electron microscope, SEM, (SEM) (model: Inspect™INSPECT F50) at different magnifications.

### 2.4 Adsorption experiments

In the batch adsorption experiment, the effect of the initial fluoride concentration (1 mg/L, 2 mg/L, 4 mg/L, 8 mg/L, and 10 mg/L), the effect of mixing time (10 min, 20 min, 40 min, 60 min, and 80 min), the effect of solution pH (5, 7, 9, and 10), and effect of adsorbent dosage (0.1 g, 0.3 g, 0.5 g, 1.0 g, and 1.5 g per 100 ml solution) was optimized. For the sorption of fluoride onto EC adsorbents, different pH ranges were reportedly used for metal-based and carbon-based adsorbents. Ranges from 2.0-12.0 [30], from 2.0 – 11.0 [31], from 2.0-10.0 [32], and 2.0–4.0 [33] were reported. Thus, the adsorption of fluoride in the present study was investigated at pH values ranging from 5.0 to 10.0. The choice of adsorbent dosage in the range of 1–10 mg/L concentration is based on the Ethiopian context, specifically, in the Rift valley, studied reports show that the maximum concentration of fluoride in Ethiopia was recorded 5 mg/L as reported by [34]. All the batch experiments were done in a single trial. The supernatants were withdrawn after a definite time interval and filtered using Whatman 1 filter paper (0.45μm) for DC analysis. The clear supernatant solution was measured using a multiparameter photometer. The de-fluoridation capacity of the adsorbent was calculated using Eq. (1) [35]. The concentration (loading) of the adsorbate in the solid phase equilibrium state (qe, mg/g) and at any time (qt, mg/g) was determined using Eqs. (2) and (3), respectively [36].

(1) $$\;Defluoridation\;capacity\;(DC)=\frac{Co-Ce}{m}V*\frac{mgF-}{g}$$

(2) $${\text{q}}_{\text{e}}=\frac{\text{V}\left({\text{C}}_{\text{o}}-{\text{C}}_{\text{e}}\right)}{\text{m}}$$

(3) $${\text{q}}_{\text{t}}=\frac{\text{V}\left({\text{C}}_{\text{o}}-{\text{C}}_{\text{t}}\right)}{\text{m}}$$

Where C_o_ and ${\text{C}}_{\text{t}}$ are the initial and final concentrations at time t of the fluoride concentration (mg/L) in the solution, respectively; C_e_ is the equilibrium fluoride concentration (mg/L), m; is the mass of the adsorbent (g), and V is the volume of the solution (L).

### 2.5 Analytical techniques

A Benchtop Multiparameter Meter, Orion™ Versa Star Pro™ pH/ISE/Conductivity/Dissolved Oxygen (VSTAR-CND VSTAR90) were frequently used for the determinations of residual fluoride ions. For all batch experiments, with the desired mixing time, the suspension was allowed to settle down for about 24 h. The sensors are modular and have integrated reference systems, and the fluoride ion-selective electrode (ISE) is fixed. Before the fluoride residue measurement is carried out, the known standard working solutions (1, 2, 4, 8, 10, and 12 ppm) were prepared from the 1000ppm fluoride ISE standard as F^−^ TISAB fluoride ISE standard, and checked for the exact fluoride concentration reading. Because the multimeter has a capacity for direct concentration measurement, the authors were not conducted calibration curve calculations, rather the precision was estimated for each concentration of fluorides. The measurement precision of the prepared standard solution concentration of fluoride has been found ±5%. The pH adjustment was carried out using buffer solutions which is important to clean up the interference ions in the solution. Then, the clear supernatant having the remaining fluoride concentration was measured in mg/L and DC has been calculated with Eq. (1). The pH measurements were performed with the same multiparameter stated above using the pH electrode.

### 2.6 pH at point-of-zero-charge (pHpzc)

Among the different methods of point of zero charge quantification, for the present work the pH drift techniques, measuring the pH values at the adsorbent-aqueous solution interface, were carried out. The pH at a point-of-zero charge of the prepared adsorbent was evaluated using laboratory-grade KCl in a concentration of 0.1 M to determine the reliability of the results. A mass of 0.3 g/100 ml of adsorbent was then weighed into each of the five conical flasks (100 ml) having pH values of 5, 7, 9, 10, and 12 by adjusting them using 0.1 M HCl/NaOH solutions. The flasks were corked and shaken using a shaker at 200 rpm for 24 h. After 24 h equilibration, the clear supernatants (20 mL) of the solutions were taken out from the conical flask into 25 mL plastic test tubes and allowed for a certain time to allow unwanted particles to settle out. Thereafter, charges on the surface of the adsorbent were quickly measured from the adsorbent-aqueous interface in the solution using a benchtop portable pH meter (PHS-3C).

### 2.7 Adsorption isotherms and thermodynamics

The adsorption isotherm, kinetic, and thermodynamic parameter values were calculated with their governing equations. Isotherm model equation of Langmuir and Freundlich were used to assure adsorption phenomenon of fluoride ions from the solution adsorbed by the prepared adsorbents, and estimating the residues in the solution. The adsorption isotherms with Langmuir and Freundlich model were governed according to Eq. (4) and Eq.(5), respectively [29].

(4) $$\frac{{\text{C}}_{\text{e}}}{{\text{q}}_{\text{e}}}=\frac{{\text{C}}_{\text{e}}}{{\text{q}}_{\text{m}}}+\frac{1}{{\text{q}}_{\text{m }}{\text{K}}_{\text{L}}}$$

(5) $${\text{logq}}_{\text{e}}=\text{logkf}+\frac{1}{\text{n}}{\text{logC}}_{\text{e}}$$

(6) Kc=CsCe=C0−CeCe

Where q_m_ is the adsorption capacity of the sorbents (mg/g), K_L_ is adsorption energy (L/g), Kf, and n are constants in the Freundlich model equation. The computed value of Kf and n are an indication of the relationship between adsorption capacity and intensity respectively. If n = 1, n > 1, and n < 1, then the sorption process would be linear, physical, or chemical, respectively. Kc is the dimensionless equilibrium constant, *Cs* is the concentration represents the ability of the adsorbent (solid-phase mg/L) to retain the adsorbate, and the extent of movement of the adsorbate within the solution, and *Ce* is the adsorbate equilibrium concentration in the liquid phase (mg/L), *Co* is the initial adsorbate concentration (mg/L). The adsorbed fluoride equilibrium concentration, onto the adsorbent, compared to the van't Hoff equation as equilibrium concentration in solution (Ce) ratio is governed by Eq. (6).

The absorption model shape is expressed with the dimensionless equilibrium factor, called a separation factor (R_L_) as shown Eq. (9) [38].

(7) $${\text{R}}_{L}=\frac{1}{1+\text{KL Co}}$$

Where: KL Langmuir constant (L/mg); and Co stands for the highest initial concentration of fluoride (mg/L).

The thermodynamic property can be used for exothermic and endothermic reactions in terms of Gibb's free energy, enthalpy, and entropy. The value of standard change Gibbs free energy, enthalpy, and entropy can be determined with Eqs. (7) and (8) [39].

(8) $$\Delta G^{o}=\Delta H^{o}-T\Delta S^{o}$$

(9) lnKc=−ΔGoRT=ΔSoR−ΔHoRT

Where: ΔG^o^ is standard change free Gibbs energy (kJ mol^−1^), $\Delta {\text{H}}^{\text{o}}$ is standard change enthalpy (J mol^−1^), $\Delta {\text{S}}^{\text{o}}$ is standard change entropy (J mol^−1^K^−1^), and R is the universal gas constant (8.314 J mol^−1^K^−1^).

### 2.8 Regeneration study

The desorption of used adsorbents (0.3 g/100 ml) was utilized again and again by first washing with distilled water and then by 0.1 M K_2_CO_3_ solution to remove the sorbed fluoride ions from the surface the adsorbent and 1% of 0.5 M HCl used to reactivate it for 1 and a half an hour reactivation time. This is an important aspect by checking its cost-effectiveness and validate the sustainability of the materials that can be used multiple times as an adsorbent for the studied pollutant removal. Centrifugation for 20 min with 4,000 rpm using a rotary centrifuge machine (SIGMA 3–18 KS), drying (for 12 h at 70 °C), and then grounding by mortar and pestle for the desired sieves size (less than 75 μm) were done. All the regeneration experiments were conducted at room temperature. Batch experiments were conducted as 1 mg/L fluoride solution with 0.3 g of the modified EC adsorbents at a pH of 5 for 20 min at 200 rpm mixing. The defluoridation capacity was evaluated up to the 5^th^ cycle reuse.

## 3 Result and discussion

### 3.1 Characterization of the prepared adsorbents

#### 3.1.1 Functional group determination

**Figure 1:**
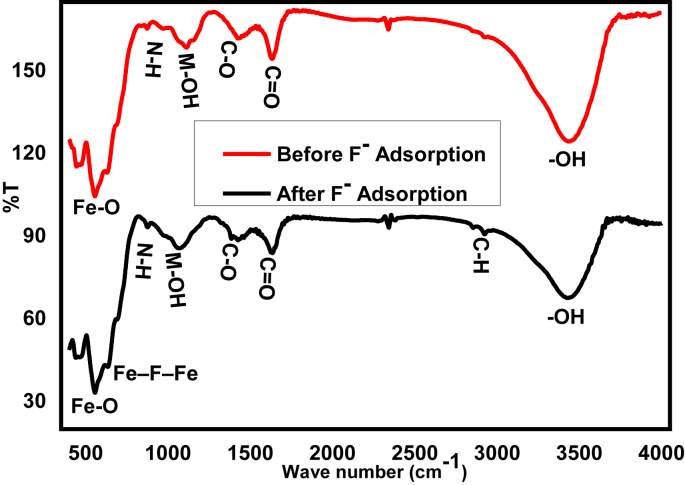
FTIR characterization of before and after fluoride adsorption on to thermally activated EC sludge.

As shown in Figure 1, the spectral peaks at 3424, and 1622 cm^−1^ were the stretching vibrations of hydroxyl functional groups from Fe_3_O_4_ and bending vibration of the carboxyl and/or carbonyl and other functional group-containing C=O, respectively. Also, the spectral peaks at 1426, and 872 cm^−1^ are attributed to the bond stretching bands of C-O stretching and N-H deformation in the amines, respectively both for before and after adsorption of fluoride ions [40]. The adsorption peaks at wavenumbers of 3439 cm^−1^ (after adsorption) are attributed to the water molecule of hydration which is similar to the reports by Feng et al, [41]. The sharp peak that appeared at 554 cm^−1^ is assigned to the Fe-O stretching vibration from Fe_2_O_3_ which the bands appear in the range of 450–650 cm^−1^ [37], representing the iron oxides are formed during adsorbent preparation through thermal treatment. The band which appears at 1098 cm^−1^ is mainly metal hydroxyl (M-OH) stretching vibration which was formed during the electrocoagulation treatments of textile effluents [42] and [43]. Also, the presence of small negligible spectral peaks located around 2918 cm^−1^ corresponds to C-H vibrations telling that there are still adsorbed organic impurities in the EC sludge [44]. After fluoride adsorbed onto the prepared adsorbent, the strong and sharp peak observed at 1426 cm^−^ becomes weaker and shifts to a low wavenumber of 1382 cm^−1^ due to the adsorbent-adsorbate interactions. Also, the small peak at 625 cm^-^1 is corresponding to Fe–F–Fe stretching after fluoride sorption. The FTIR spectra confirmed that a new peak at 625 cm^−1^ is the Fe –F–Fe stretching after fluoride sorption through electrostatic attraction. This is matched with the analysis reported by Vasudevan et al., [45]. Thus fluoride can have a capacity to link with the prepared iron-based adsorbents as a complex form and have a tendency to precipitate for fluoride adsorption.

#### 3.1.2 Diffraction pattern analysis

**Figure 2:**
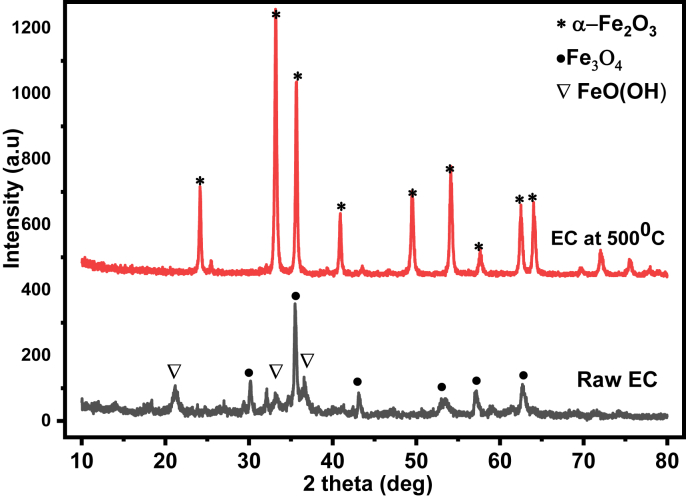
X-ray diffraction pattern of raw and thermally activated at 500 °C electron-coagulated (EC) sludge adsorbent.

The diffraction patterns of untreated and thermally activated EC sludge adsorbents are shown in five main characteristic peaks from the diffractograms as shown in Figure 2. The pattern at 2θ = 30.2°, 33.4°, 35.9°, 40.7°, 43°, 49.7°, 54.2°, and 62.3° suggests that the structure of goethite and magnetite from the raw EC sludge [46]. However, the diffraction pattern around at 2θ = 24.08°, 33.23°, 35.69°, 40.97°, 49.57°, 54.08°, 57.64°,62.48°, and 64.09° are the characteristics of hematite (α-Fe_2_O_3)_ structure and the intensity becomes sharpen for thermally treated (at 500 °C) EC sludge due to oxidative formations of α-Fe_2_O_3_ from iron oxy-hydroxide contained textile sludge [47]. Comparatively, the intensity and crystalline nature in the thermally treated EC sludge at 2θ = 30.2°, 33.3°, and 35.7° is higher than the raw one. After thermal treatment, the diffraction pattern at 2θ = 21.3° in raw EC sludge present in iron oxy-hydroxide and goethite (FeOOH) disappeared completely with transforming to hematite at 500 °C. From the diffraction patterns, it can be deduced that the raw EC contained dominantly goethite and magnetite but after thermal treatment, they were completely transformed into hematite.

#### 3.1.3 Surface morphology analysis

**Figure 3:**
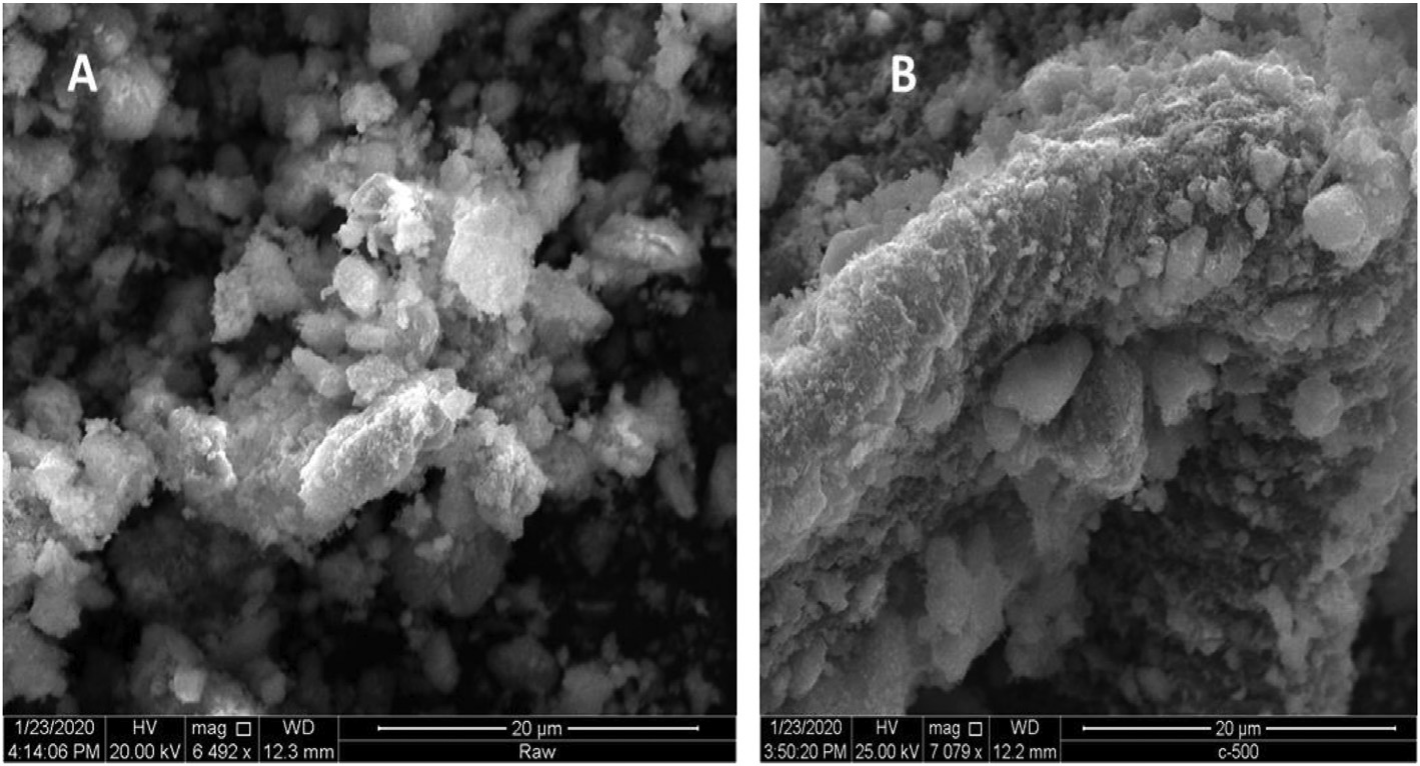
SEM Micrographs of (A) raw (unactivated), (B) thermally treated at 500 °C electro-coagulated sludge.

The surface morphology of untreated (raw) and thermally activated (at 500 °C) EC sludge is shown in Fig 3a and b, respectively. The morphological structure is shown as an uneven edge agglomerate and adsorptive nature for thermally treated EC sludge but the raw one is observed as an open-ended fog-like structure in nature and formed a complex structure. The micrographs for raw EC sludge are observed less rough than thermally treated which has a similar characteristic with the pure iron oxide powders [48]. The higher disordered/amorphous surface is observed in the raw EC sludge, filling with several small scraps of other magnetite iron oxides and goethite (FeO(OH)) resulting in a complex surface structure. But, the thermally activated EC sludge showed rougher and agglomerated surfaces indicating that the volatile organic matter may remove from the bulk sludge samples and transformed into a pure and high crystalline powder. This can make the adsorbent surfaces more adsorptive morphology. This is also further confirmed by X-ray diffraction as shown in Figure 2 in that the produced powder is dominantly the maghemite (γ-Fe_2_O_3_) and hematite (α-Fe_2_O_3_) type of iron oxides [29]. Thus, it can be deduced that the goethite and magnetite materials underwent an oxidative transformation of those nanoparticle iron species.

Moreover, the agglomeration of the nano-sized particles can not be avoided as shown in Figure 3 in that the micrograph at 20 μm exhibit irregularly shaped nanoparticles. Reports confirmed that iron oxide nanoparticles exhibit mainly spherical if they can be synthesized from the pure iron precursors [49]. However, the iron particles in this study high aggregation and non-pure spherical shapes are not observed. This may be due to other metals that could exist in-situ the iron oxide powders because of the non-use of pure iron agents as a precursor. In addition, the rough surface in the topography of the prepared adsorbent was beneficial for their dispersion to make composites sorbents with materials for the enhanced removal of fluoride ions. Figure 3 (B) showed the presence of pores on the surface edge tells that it was beneficial for the adsorption process of fluoride ions. Remarkably, the thermally activated EC sludge (Figure 3 B) surfaces look much tighter and agglomerated hills, as compared with the unactivated (Figure 2A), suggested that the adsorbent powder was formed as a crystalline and relatively pure materials. This is also confirmed by scholars who researched the elemental composition of EC sludge, using an iron electrode, with iron species is about 83% as reported by Castañeda-DÍaz et al [43] out of different elements that have been recorded. Thus, it is confirmed that the surface morphology of this study was reported as a similar structure with pure iron-based nanoparticles including hematite [50].

### 3.2 Batch adsorption experiments

#### 3.2.1 pH at point-of-zero-charge (pHpzc)

**Figure 4:**
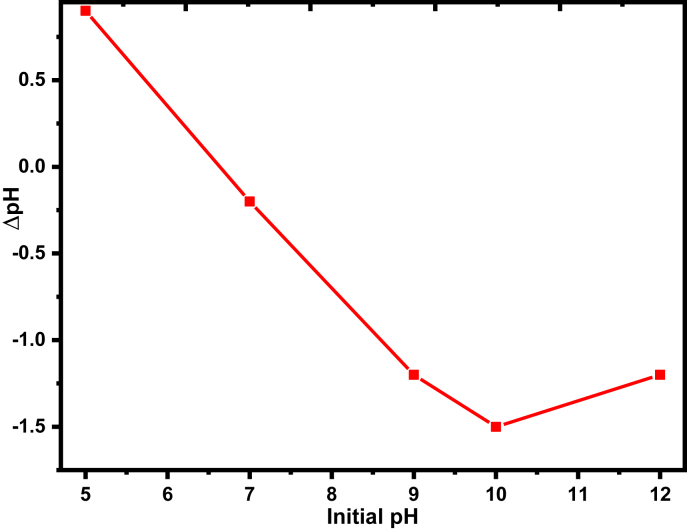
pH at a point-of-zero charge of the thermally treated adsorbent for defluoridation using 0.1 M M KCl solution.

The adsorption phenomenon from the solid-liquid phase is affected by the interfacial electrostatic properties of the adsorbent. The point of zero charges (ZPC) is a very important parameter that can validate the electrical nature of the adsorbent in the solution electrolyte interface. Particularly, for the metal-based oxides, the PZC is the pH at which the adsorbent surface charge becomes zero. If there are no adsorbed ions other than the proton and hydroxyl, the PZC accords with the isoelectric point [51]. The change in pH (Δp*H* = pH _*f*_ - pH _i_) electrokinetic behaviors of the prepared adsorbents were determined and plotted against the initial pH as shown in Figure 4, and the pHpzc was found as 6.64. Solutions pH over headed the pHpzc tells that the prepared adsorbents are negatively charged and becomes positively charged at a pH lower than pHpzc.

#### 3.2.2 Effect of mixing time

**Figure 5:**
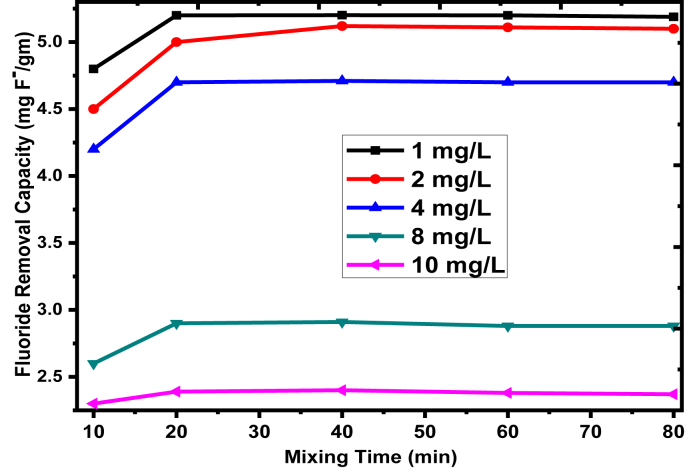
Effect of contact time on the fluoride removal capacity of the, 1–10 mg/L initial fluoride concentration (with experimental conditions: pH = 5.0, adsorbent dosage = 0.3 g/100 ml, at the temperature of 25 ± 3 °C, and rpm of 200).

The DC experiment was investigated to determine the adsorption capacity of the adsorbent from NaF solution with experimental conditions of mixing time from 10 to 80 min with 100 mL of 1–10 mg/L as initial fluoride concentration, pH of 5.0, and adsorbent dosage of 0.3 g/100 ml at ambient temperature (25 ± 3 °C). The adsorption capacity of the adsorbent towards fluoride ion removal at different mixing times was determined and presented in Figure 5. The adsorbed fluoride ion from the solution was increased significantly with increasing mixing time from 10 to 20 min. However, further increasing of the mixing time did not increase the adsorbents removal capacity, telling that the equilibrium removal capacity was reached after 20 min. It has shown that the optimum mixing time for adsorption of fluoride by the prepared adsorbent was 20 min at a given fluoride concentration, solution pH, and adsorbent dosage. The maximum fluoride removal capacity obtained was 5.12 mgF^-^/g at the equilibrium time and a concentration of 1 mg/L as optimum conditions. From this, it can be deduced that the most active sites from the adsorbent surface are enough for the adsorption of sorbate at the initial stage. However, the remaining active sites on the adsorbent surface become hard to be adsorbed as time goes by due to repulsive forces between the sorbates in the liquid phase onto the solid phase [52].

#### 3.2.3 Effect of solution pH

**Figure 6:**
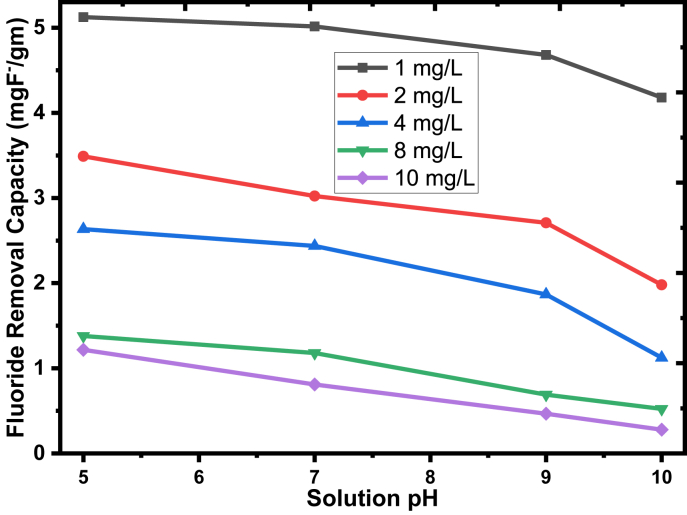
Effect of solution pH on the fluoride removal capacity of the sorbents with 1–10 mg/L (experimental conditions: 0.3 g/100 ml of adsorbent dosage, at a mixing time of 20 min, the temperature of 25 ± 3 °C, and rpm of 200).

For the removal of fluoride from an aqueous solution through adsorption, pH is considered to be a significant parameter that can affect the sorbent capacity. The effect of pH on the defluoridation onto the adsorbents from the solution was determined at five different initial fluoride concentrations ranging from 1 to 10 mg/L with an adsorbent dose of 0.3 g/100 ml, mixing time of 20 min, and temperature of 25 ± 3 °C. The solution pH was adjusted using 1.0 M solutions of sodium hydroxide and hydrochloric acid. The effect of pH on defluoridation by the prepared adsorbent from EC sludge is shown in Figure 6. The fluoride removal capacity sharply increases as fluoride ion concentration decreases. Also, the fluoride removal capacity linearly decreases as the pH value increases from 5 to 9, and sharply decreases up to pH 10, particularly on the low concentrations of fluoride. The fluoride removal capacity of 5.12, 3.49, 2.63, 1.38, and 1.22 mg of F^−^/g were recorded at 1, 2, 4, 8, and 10 mg/L initial fluoride concentration, respectively at pH 5, 0.3 g/100 ml of adsorbent dosage, mixing time of 20 min, and temperature of 25 ± 3 °C. Thus, the equilibrium value was found at a pH of 5.0 to get maximum adsorption capacity. However, above pH 5, the removal capacity decreases as the solution pH increases. Low removal capacity at basic media is due to the increase of hydroxyl ion in the solution suggesting that the competition between hydroxyl ion and fluoride ion from the solution produces electrostatic repulsion [53].

A similar study on the fluoride adsorptions by iron oxide adsorbents prepared from waste sludge was reported with the optimum adsorption capacity of 20 mg/g at pH 4.0, and the treatment temperature was at 300 °C which is mainly maghemite iron oxide [30]. The basic difference with the present study was the treatment temperature at 500 °C produced hematite phase iron oxide. The number of adsorption sites increases with the formation of nanopores when iron ox hydroxide transforms to Fe_2_O_3_ up to 300 °C. But, above this treatment temperature, a shrinkage of nanopores could occur and is resulted in a decrease in this nanopore, eventually, the adsorption capacity is decreased.

#### 3.2.4 Effect of adsorbent dosage

**Figure 7:**
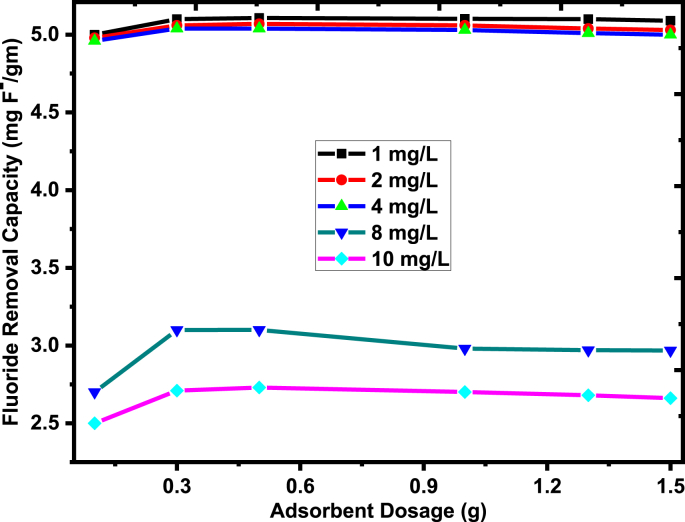
Effect of adsorbent dosage on the fluoride removal capacity of the sorbents with 1–10 mg/L (experimental conditions: pH of 5, mixing time of 20 min, 25 ± 3 °C temperature, and rpm of 200).

The effect of adsorbent dose on fluoride removal capacity was determined at 0.1 g, 0.3 g, 0.5 g, 1.0 g, and 1.5 g per 100 ml of adsorbent with 1–10 mg/L initial fluoride concentration while keeping other parameters constant (solution pH of 5 at 20 min contact time and 25 ± 3 °C temperature). As shown in Figure 7, the removal capacity of fluoride dramatically increases for all initial fluoride concentrations as the adsorbent dose increases from 0.1 g/100 ml to 0.3 g/100 ml. Further, an increase in an adsorbent dose up to 1.5 g/100 ml did not increase the fluoride removal capacity and leads to an equilibrium condition with a slight decline. This is due to the fact that an increase in adsorbent dose leads to an increase in surface area and provides many active sites for the sorption of fluorides and also the attainment of equilibrium is due to the saturation of the sorbent active site in solution [54]. As can be observed in Figure 7, the fluoride adsorption capacity of the adsorbent is high for fluoride concentrations of 1, 2, and 4 mg/l. Whereas the increase of initial fluoride concentration from 4-10 mg/L, the adsorption capacity was drastically declined. The reason behind this is that the total quantity of contact surface of the prepared adsorbent to the fluoride ions in the solution has been played a vital role in the adsorption phenomenon. That means, the availability of excess adsorption sites than that of the sorbents, assuming that the number of adsorption per unit mass of adsorbents remains constant. This is due to the increase in surface area and the availability of more sites for the adsorption of fluoride [55]. In the present study, the optimum adsorbent dosages were found to be 0.3 g/100 ml with the fluoride removal capacity of 5.12 mgF^-^/g for 1 mg/L of fluoride ions at a pH of 5, and mixing time of 20 min.

### 3.3 Adsorption isotherm

The Langmuir isotherm model designates the adsorption at the solid-liquid interface that empirically assumes the phenomenon is the monolayer adsorption. That means the adsorption occurs at the definite sites of the adsorbent and represented the monolayer capacity of the prepared materials. However, the Freundlich isotherm model describes the non-ideal and reversible adsorption processes and most appropriate to the use of heterogeneous (multilayer) adsorbent materials which defines the surface heterogeneity and active site in the adsorption phenomenon that occurs [56].

Equilibrium adsorption isotherm models for the fluoride removal capacity study were investigated using Langmuir and Freundlich linear models [57] and the calculated values are shown in Table 1. Adsorption isotherms of fluoride removal capacity on prepared adsorbent at a temperature of 298, 313, 323, and 353 K were studied. Freundlich plots were used to calculate Kf, n, and 1/n isotherm parameters from the slope and intercept, respectively as shown in Table 1. Isotherm parameters, 1/n, and n values are indicated between 0 to l and 1 to10, respectively suggested that the adsorption of fluoride on prepared adsorbent is favorable. In the Freundlich adsorption model, the K_f_ value increases with increasing temperature telling that the adsorption process is endothermic. It is found that from the correlation coefficient (R^2^) values that the equilibrium data of the process was well fitted by the Langmuir model as compared to the Freundlich model suggests that the homogenous adsorption processes have occurred. That means each of the fluoride ions owns constant enthalpies and sorption activation energy, and it is the physisorption phenomenon that is dominated. Moreover, the correlation coefficient of the Langmuir model is greater than that of Freundlich, showing the Langmuir sorption modeling isotherm describes more accurately the sorption of fluoride adsorption on the prepared adsorbents. Thus, the Langmuir isotherm fits the experimental data appropriately due to the homogenous and uniform distribution of active sites on prepared adsorbents.

**Table 1:** Langmuir and Freundlich adsorption isotherm parameters for adsorbent prepared from EC sludge.

Temperature (K)	Langmuir isotherm				Freundlich Isotherm			
	Q_m_ (mg/g)	R^2^	K_L_ (L/mg)	R_L_	1/n	n	K_f_(mg/g)	R^2^
298 K	4.45	0.997	2.96	0.36	0.561	2.91	5.53	0.9995
313 K	4.66	0.9990	3.59	0.41	0.615	3.03	5.75	0.996
333 K	4.71	0.9999	4.02	0.51	0.646	3.41	6.13	0.988
353 K	4.77	0.9998	4.60	0.61	0.675	3.92	6.43	0.967

**Figure 8:**
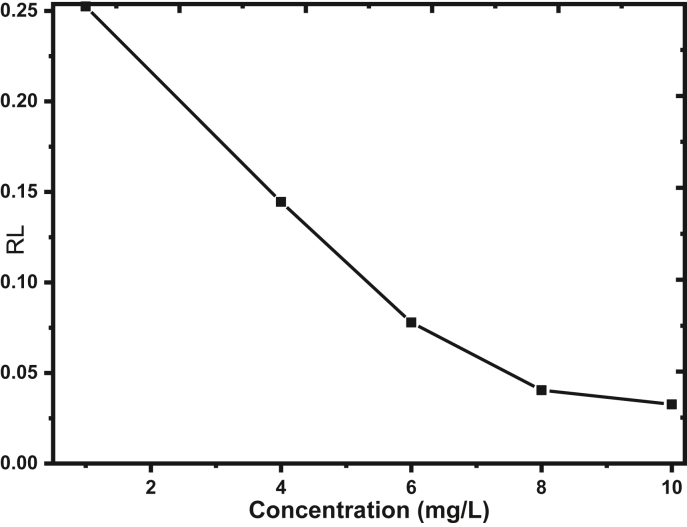
Separation factor versus fluoride concentration plot.

Moreover, the calculated RL value for electro-coagulated sludge was found that 0.0327 at the highest (10 mg/L) of fluoride concentration that is in the range of 0 and 1 tells that the adsorption phenomenon is favorable. The RL value degreases as the fluoride concentration increases as shown in Figure 8 suggests that the adsorption favorability goes to the irreversible isotherm type of adsorption.

### 3.4 Thermodynamic parameters

Standard free energy change $\left(\Delta {\text{G}}^{\text{o}}\right)$, standard change in enthalpy $\left(\Delta {\text{H}}^{\text{o}}\right)$, and standard change in entropy $\left(\Delta {\text{S}}^{\text{o}}\right)$ are the basic thermodynamic parameters that are influenced within the adsorption processes and can be calculated as reported by Khan and Singh [58]. As shown in Table 2, the disorder and endothermic behavior in the fluoride removal capacity phenomenon onto the prepared adsorbent were confirmed by the positive values of $\Delta {\text{S}}^{\text{o}}$ and $\Delta {\text{H}}^{\text{o}}$. Fluoride adsorption onto the prepared adsorbent has shown negative values of $(\Delta {\text{G}}^{\text{o}}$ from 298 K to 353 K temperature ranges with increasing negativity. Gibb's free energy value comes out to be positive which suggests being in a non-spontaneous reaction. The small value of Gibb's free energy shows the adsorbents' higher adsorption potential. The positive value of ΔH^o^ suggests that the process is endothermic. At a lower temperature, the smaller values of ΔG^o^shows the higher amount of fluoride adsorption onto the adsorbent's surface.

**Table 2:** Adsorption thermodynamic parameters of prepared adsorbent from EC sludge.

Adsorbent	$\Delta {\text{G}}^{\text{o}}\left[\text{KJ}.\text{K/mol}\right]$				$\Delta {\text{H}}^{\text{o}}\left[\text{KJ.K/mol}\right]$	$\Delta {\text{S}}^{\text{o}}\left[\text{J.K/mol}\right]$
	Temperature (K)					
	298	313	333	353		
EC Sludge	-3.90	-3.27	-2.52	-1.97	14.2	49.4

### 3.5 Adsorption kinetics studies

The adsorption kinetics of the fluoride removal phenomenon onto the prepared adsorbent was computed by using pseudo-first-order and pseudo-second-order models. To test those models, a set of experimental data were employed. Besides, the values kinetic parameters were computed as summarized in Table 3. It is essentials that the kinetic models can confirm the experimental data and model-predicted values during the adsorption phenomenon. The relatively higher value demonstrates the more favorable model equation to the kinetics of fluoride adsorption onto the prepared adsorbents. The Pseudo-first-order rate is governed by Eq. (10) with the boundary conditions t = 0 to t = t and qt = 0 to qt = qt, it becomes Eq. (11). The result of the pseudo-second-order model is shown in Eq. (12) after the integration of the linearized form of Eq. (13) [59]. The value of k_2_ (g/mg min) and qe was computed from the slope and intercept of the $\frac{dqt}{\text{qt}}$ versus t [35].

(10) $$\frac{\text{dqt}}{\text{dt}}=\text{k}1\left(\text{qe}-\text{qt}\right)$$

(11) $$\text{log}\left(\text{qe}-\text{qt}\right)=\text{logqe}-\left(\frac{\text{k}1}{2.303}\right)$$

(12) $$\frac{\text{dqt}}{\text{dt}}=\text{k}2\left(\text{qe}-\text{qt}\right)2$$

(13) $$\frac{\text{t}}{\text{ qt}}=\frac{1}{{\text{k∗}}_{2}{\text{qe}}^{2}}+\left(\frac{1}{\text{qe}}\right)\text{t}$$

Where, *Qe* (mg/g) is the amount of the fluoride adsorbed at equilibrium, *qt* (mg/g) is the amount of fluoride adsorbed at a particular time t (min) and k_1_ (min^−1^) *is* the rate constant of adsorption pseudo-first-order adsorption. To obtain the rate constant, the value of log (qe-qt) was linearly correlated with time (t). The value of k_1_and predicted qe has been computed from the slope and intercept of the plot. The correlated coefficient, R^2^ of the pseudo-second-order kinetic model is higher than pseudo-first-order kinetics models as shown in Table 3. The values of the pseudo-first-order rate constant (k1) 0.0072, 0.0591, 0.0042, and 0.0025 min-1 for the 298, 313, 333, and 353 k, respectively. And the values of the pseudo-second-order rate constant (k2) were 0.0995, 0.0832, 0.1024, and 0.0063 g/mg∗min for the 298, 313, 333, and 353 k, respectively. This tells that the adsorption phenomenon with the prepared adsorbent is seemed to be employed with both of the kinetic models, and the batch adsorption experiment for the removal of fluoride by the prepared adsorbent favors more to the pseudo-second-order kinetic processes. Because the value of the correlation coefficient (R2 = 0.9999) is high for Pseudo-Second-order kinetic model as compared to that of the Pseudo-first-order (R2 = 0.9889), and best fits with the Pseudo-Second-order model.

**Table 3:** Pseudo-first-order and Pseudo-Second-order adsorption kinetics computed parameters at different adsorption temperature.

Parameters	**Pseudo-first-order**			
	Temperature (K)			
	298	313	333	353
Q_e_ (mg/g)	3.25	2.75	3.02	3.35
K_1_ (min^−1^)	0.0072	0.0591	0.0042	0.0025
R^2^	0.9899	0.9675	0.9889	0.9789
	**Pseudo-Second-order**			
Q_e_ (mg/g)	4.13	3.82	3.65	3.92
K_2_ (g/mg∗min)	0.0995	0.0832	0.1024	0.0063
R^2^	0.9998	0.9998	0.9999	0.9997

Moreover, the calculated adsorption capacities Qe (mg/g) in the Pseudo-Second-order kinetic processes is higher than Pseudo-first order kinetics implies that the Pseudo-Second-order kinetic favors more for the adsorptions of fluoride ions on to prepared adsorbents. In principle, adsorbate-adsorbent interaction depends on the specific areas and pore volume values increase/decrease of the adsorption capacities of the sorbents that can be controlled by the mass transfer.

Thus, the results of the present study suggested that the Pseudo-Second-order kinetic model could be regarded as more followed by the prepared adsorbents for the removal of fluoride. This means that the model was more practical to confirm the experimental data with the predicted values in the adsorption process.

### 3.6 Regeneration and number of cycles

**Figure 9:**
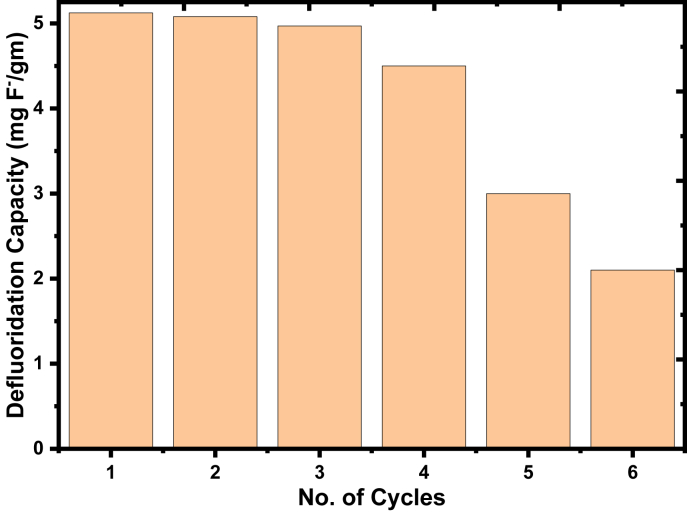
Performance of the adsorbent for the removal of fluoride after regeneration and reuse.

The regeneration and reuse of the prepared adsorbents for fluoride removal capacity were done up to six sequential cycles as shown in Figure 9. The fluoride removal capacity of the adsorbent is decreased from 5.124 mgF^-^/g to 2.1 mgF^-^/g with continuous regeneration up to the 6^th^ cycle of reuse. Regenerated adsorbent for fluoride removal capacity was recorded as best for the first three cycles and decreased dramatically for the 5^th^ and 6^th^ cycles. This suggests that the reuse of this adsorbent is efficient up to the subsequent 4^th^ cycle for fluoride removal which means that the adsorbent reduced fluoride concentration to the lowest acceptable level, after reusing. From the sharp decrease for the 5^th^ and 6^th^ cycles, it can be deduced that the fluoride ions could not further be adsorbed onto the adsorbents due to lack of enough active site through collapsing, eventually, repulsion with each other may occur [60].

### 3.7 Comparison of defluoridation between the prepared adsorbents with other reported adsorbents

The outcomes of this study were compared with numerous other adsorbents are found in Table 4. From a previous study, it was found that the existent study indications acceptable removal values in comparison to other research discoveries. The fluoride removal capacity in the present study was varied over some of the other adsorbents. This is due to the different experimental conditions (initial fluoride concentration, solution pH, sorbent dosages, and other parameters. From the present comparison analysis, the pH value was taken as a basic influential parameter. Moreover, the adsorption capacity recorded is mostly varied due to the adsorbent modification methods, such as the chemical, thermal, or combination were conducted to modify the adsorbents that influence the removal capacity. However, in the present study, only thermal treatment techniques were employed to prepare the materials from EC sludge, hence it is advantageous compared with those presented in previous works in the cost-effectiveness perspective.

**Table 4:** Comparison of fluoride adsorption removal capacity of the various adsorbent.

Adsorbents	Removal (mg/g)	Optimum operating parameters					Reference
		Concentration	pH	mixing time	Dose	Modified/raw adsorbent	
Sludge waste from electro-coagulation	45.5 mg/g	25 mg/l	6.0	120 min	4 g/l	Thermally activated	[61]
iron–aluminum nanocomposite	42.95 mg/g	10 mg/L	5.5	120 min	0.25 g/L	Chemically activated	[62]
Polypyrrole/Fe_3_O_4_	17.6 mg/g	10 mg/L	6.5	20 min	0.200 g	Chemically activated	[63]
Rice husk activated carbon	7.9 mg/g	13 mg/L	7.0	180 min	5 g/L	Chemically activated	[64]
Corn activated carbon	5.8 mg/g	13 mg/L	5.5	300 min	4 g/L	Chemically activated	[64]
Tea-Al-Fe bio-sorbents	18.52 mg/g	10 mg/L	7.0	120 min	2 g/L	Calcined and Chemically activated	[65]
Corn Stover biochar (CSBC)	6.42 mg/g	10 mg/L	2.0	120 min	5.0 g/L	Pyrolysis and Chemically activated	[66]
Pinewood biochar	7.66 mg/g	10 mg/L	2.0	48 h	10 g/L	activated with pyrolysis	[67]
Activated carbon of *Catha edulis*	18 mg/g	30 mg/L	2.0	60 min	15 g/L	Thermally and chemically activated	[68]
Monetite	6.4 mg/g	25 mg/L	7.0	24 h	4 g/L	chemically activated	[69]
Fired Clay Pots	1.6 mg/g	10 mg/L	4.5	10 min	1 g/L	Raw	[70]
Brewery wasted diatomite	0.617 mg/g	10 mg/L	5.0	30 min	60 g/L	Acid activated	[71]
Natural grade diatomite	0.917 mg/g	10 mg/L	5.0	60 min	60 g/L	Acid activated	[71]
Iron oxide	60.8 mg/g	50 mg/L	6.5	2 min	1 g/L.	Chemically modified and Ethanol treated	[72]
Fe_3_O_4_/Al_2_O_3_ Nanoparticles	70.4 mg/g	10 mg/L	7.0	20 min	1 g/L	Sulfate-doped	[73]
Iron Oxide-Hydroxide Nanoparticles	16.7 mg/g	10 mg/L	7.28	180 min	1 g/L	Facile synthesized	[31]
waste iron oxide	20.4 mg/g	6 mM	4.0	48 h	1 g/L	Thermally activated	[30]
Electro-Coagulated Sludge	5.124 mg/g	1 mg/L	5.0	20 min	3 g/L	Thermally treated	Present study

## 4 Conclusion

Nowadays, the preparation and utilization of the non-conventional adsorbent from different wasted materials for different contaminant removal from water and wastewater are attracting attention due to their cost-effectiveness, versatility simplicity. For example, from biomass, natural clays, and sludges for wastewater treatment plants (the present study). Iron-based adsorbents were prepared (with beneficiation, and thermal activation) from EC sludge in the wastewater treatment to utilize for the fluoride ions removal in aqueous solutions. The fluoride iron-containing solutions were prepared using reagent-grade sodium fluoride (NaF).

The characterization of prepared adsorbent signifying that dominantly iron oxide (hematite) is contained after thermal activation of the EC sludge. The prepared adsorbents were shown optimum DC values of 5.12 mgF-/g with experimental conditions: mixing time = 20 min, adsorbent dosage = 0.3 g/100 ml, initial fluoride concentration = 1 mg/L, and pH = 5 at the temperature of 353 K. Defluoridation studies onto the prepared adsorbents are competitive with pure iron-based oxide and/or hydroxides used as an adsorbent. The adsorption of fluoride ions fits more on the Langmuir isotherm model, Pseudo second-order kinetics, and is thermodynamically spontaneous.

The utilization of EC sludge for different applications, such as contaminant removal is effective low-cost, and bi-functional as environmental management strategies, “waste-to-valuable products”. The study revealed that the thermally activated adsorbents from EC sludge were found as a promising alternative for fluoride removal at the employed experimental conditions. The cost estimation including the manpower, operation and machinery costs in the utilization of waste EC sludge as an alternative adsorbent with simple preparation techniques should be researched together with its sustainability. Also, different ionic chemicals existence with the fluoride ion in the natural water for efficiency of the adsorption capacity should be evaluated.

## Declarations

### Author contribution statement

Tadele Assefa Aragaw: Conceived and designed the experiments; Performed the experiments; Analyzed and interpreted the data; Contributed reagents, materials, analysis tools or data; Wrote the paper.

### Funding statement

This research did not receive any specific grant from funding agencies in the public, commercial, or not-for-profit sectors.

### Data availability statement

Data will be made available on request.

### Declaration of interests statement

The authors declare no conflict of interest.

### Additional information

No additional information is available for this paper.
